# Incidences of Rocuronium Use during Anesthetic Induction in Adult Patients Undergoing Orthopedic Surgery Using Supraglottic Airway Devices: A Retrospective Analysis

**DOI:** 10.3390/jcm13175299

**Published:** 2024-09-06

**Authors:** Yu-Kyung Bae, Hyo-Seok Na, Jung-Won Hwang, Young-Jin Lim, Sang-Hwan Do

**Affiliations:** 1 Department of Anesthesiology & Pain Medicine, Seoul National University Bundang Hospital, Seongnam 13620, Republic of Korea; vansuri27@gmail.com (Y.-K.B.); hsknana@gmail.com (H.-S.N.); jungwon@snubh.org (J.-W.H.); 2 Department of Anesthesiology & Pain Medicine, Seoul National University College of Medicine, Seoul 03080, Republic of Korea; limyjin@snuh.org

**Keywords:** supraglottic airway devices, rocuronium, anesthetic induction, propofol, remimazolam, inhalation agent, midazolam, orthopedic surgery

## Abstract

**Background/Objectives**: Neuromuscular blocking agents (NMBAs) are not usually necessary during the induction of general anesthesia in patients using supraglottic airway (SGA) devices. In this study, we assessed the incidences of rocuronium use in adult patients undergoing general anesthesia using SGA devices. **Methods**: From September 2022 to August 2023, the medical records of adult patients (≥19 years) who underwent orthopedic surgery using SGA devices were retrospectively investigated. The incidences of rocuronium use during anesthetic induction were analyzed according to the anesthetic induction drug. The association of rocuronium use during anesthesia was analyzed in terms of demographic (age, sex, height, and weight), surgical (surgical time), and anesthetic factors (premedication, anesthetic agent, anesthetic time). **Results**: In total, 321 patients were enrolled. The incidence rate of rocuronium use during anesthetic induction was 28.3%. In the subgroup analysis, patients receiving total intravenous anesthesia (TIVA) with propofol (PPF) and remifentanil showed a markedly lower incidence (14.4%) than the other anesthetic groups. Premedication or short anesthetic duration was associated with lower incidences of rocuronium use. Demographic and other anesthetic factors did not seem to affect the incidences of rocuronium use during anesthesia. **Conclusions**: The incidence of rocuronium use during anesthetic induction with SGA devices was significantly lower with the PPF-TIVA compared to that using remimazolam-TIVA or inhalational anesthesia. Premedication with midazolam and shorter operation times were associated with a significantly lower incidence of rocuronium use.

## 1. Introduction

Supraglottic airway (SGA) devices have been widely used for general anesthesia in short surgical procedures. Compared to endotracheal tubes, SGA devices lead to fewer hemodynamic changes during device insertion and no irritation to the trachea, which can cause postoperative sore throat [1]. An outstanding advantage of SGA devices is that they can secure the airways without the need for neuromuscular blocking agents (NMBAs) [2]. SGA devices without NMBAs have been used less invasively and more effectively than the endotracheal tube; however, they also require adequate suppression of airway reflexes, which may otherwise lead to incorrect or failed SGA device placement [3]. During anesthetic induction, neuromuscular blockade not only fails to facilitate SGA insertion [4], but also does not reduce pharyngolaryngeal trauma or discomfort after surgery [5]. Nonetheless, there may be instances in which NMBAs are required during anesthetic induction using SGA. For example, neuromuscular blockade would be helpful if mask ventilation was not satisfactory in the early phase of anesthetic induction even before SGA insertion. In addition, NMBAs may be also required after SGA insertion if ventilation was not satisfactory or the patient showed hiccups or spontaneous breathing. Therefore, in this retrospective study, we aimed to investigate the incidences of rocuronium use, the representative non-depolarizing NMBA, according to various anesthetic factors during anesthetic induction using SGA devices.

## 2. Materials and Methods

### 2.1. Study Population and Ethical Approval

The study protocol was approved by the Institutional Review Board (IRB) of Seoul National University Bundang Hospital (approval no. B-2401-875-104). The IRB waived the requirement to obtain informed consent from the patients due to the retrospective nature of this study.

The medical records of patients aged 19 years and older with an American Society of Anesthesiologists physical status of I–III who were scheduled to use SGA devices for airway management to undergo minor orthopedic surgery from September 2022 to August 2023 were retrospectively reviewed. Patients who underwent endotracheal intubation for airway management and those who switched from spinal anesthesia to general anesthesia were excluded. Cases where the anesthetic agent was changed during anesthetic induction were also excluded.

### 2.2. Anesthetic Management

For patients with anxiety, 0~3 mg of IV midazolam was administered in the reception area. Basic monitoring, including electrocardiography, non-invasive blood pressure measurement, pulse oximetry, and bispectral index measurement (BIS, Medtronic, Minneapolis, MN, USA), was performed in the operating room.

Anesthesia was induced using three different methods. In the propofol (PPF)-total intravenous anesthesia (TIVA) group, PPF 4 µg/mL was administered and remifentanil up to 4 ng/mL was administered by a target-controlled infusion method during the induction of anesthesia, and then PPF was maintained at a rate of 2.5~3.5 µg/mL during surgery. In the remimazolam (RMZ)-TIVA group, RMZ (Byfavo^®^ Inj., Hana Pharm Co., Ltd., Seoul, Republic of Korea) was administered at 6 mg/kg/h, and remifentanil up to 4 ng/mL was administered by the target-controlled infusion method during the induction of anesthesia, and then RMZ was maintained at a rate of 1~2 mg/kg/h during surgery. In the inhalation group, 1.5~2 mg/kg PPF and remifentanil were administered via target-controlled infusion at an effect-site concentration of up to 4 ng/mL. Anesthesia was maintained using 1~1.5 MAC of inhalation anesthetic (sevoflurane or desflurane). Nitrous oxide was not used. Remifentanil up to 3 ng/mL was administered by the target-controlled infusion method during the maintenance of anesthesia in all three groups.

The SGA device (LMA^®^ Flexible Teleflex; Athlone Co. Westmeath, Ireland, I-gel^®^; Intersurgical, Crane House, Molly Millars Lane, Wokingham, Berkshire, UK, LMA^®^ Supreme Teleflex; Athlone Co. Westmeath, Ireland) was inserted at the discretion of the attending anesthesiologist when patients were fully sedated. SGA insertion was initiated when BIS fell below 60. In all of the patients, no NMBAs were used on a routine basis during induction of general anesthesia. In our institution, the administration of NMBAs during anesthetic induction using SGA devices is totally left to the discretion of the attending anesthetist. There are several tentative guidelines in place in our department, as described below: If necessary, 0.2~0.4 mg/kg of rocuronium was administered under the following situations: when the peak inspiratory pressure went over 30 cmH_2_O; when the capnography showed a significant obstructive pattern; when the mouth did not open adequately during insertion of SGA device; or when spontaneous breathing, hiccup, gagging, coughing, or laryngospasm occurred and persisted after SGA insertion and during surgery [6]. The reasons for using rocuronium during anesthetic induction could be inferred retrospectively from the anesthetic record.

After the successful insertion of the SGA, the patients were mechanically ventilated. Ventilation parameters were set as follows: tidal volume 6 to 8 mL/kg, respiratory rate 10 to 15 breaths/min, fresh gas flow 2.5 L/min, inspired oxygen fraction 50%, and positive end-expiratory pressure 5 cmH_2_O if necessary, maintaining end-tidal carbon dioxide pressure at 33 to 38 mmHg.

At the end of the surgery, the anesthetics were discontinued and RMZ or rocuronium was reversed with flumazenil or neostigmine and sugammadex, respectively. After confirming the return of spontaneous breathing, the SGA device was removed. The patients were then transferred to the post-anesthesia care unit. When the post-anesthetic recovery score reached 9 or higher, the patients were discharged from the post-anesthesia care unit.

### 2.3. Date Collection and Statistical Analysis

Data on sex, age, height, weight, operation and anesthesia time, use of midazolam at the reception area, NMBAs during anesthetic induction and surgery, and SGA device type were collected.

All data were analyzed using SPSS software (ver. 20.0; IBM Corp., Armonk, NY, USA). For demographic data, Chi-square or Fisher’s exact tests and *t*-tests were used. Chi-square cross-tabulation was used to identify the differences in the incidences of rocuronium use between the anesthesia groups. A one-way analysis of variance (ANOVA) was performed among three groups to analyze the dose of rocuronium. Logistic regression was performed to analyze the relationship between the incidence of rocuronium use and various factors, such as age, sex, height, weight, body mass index, operating and anesthesia times, use of midazolam, and anesthesia group. The data are presented as means ± standard deviation or as numbers and percentages. Statistical significance was set at *p* < 0.05.

## 3. Results

**Figure 1:**
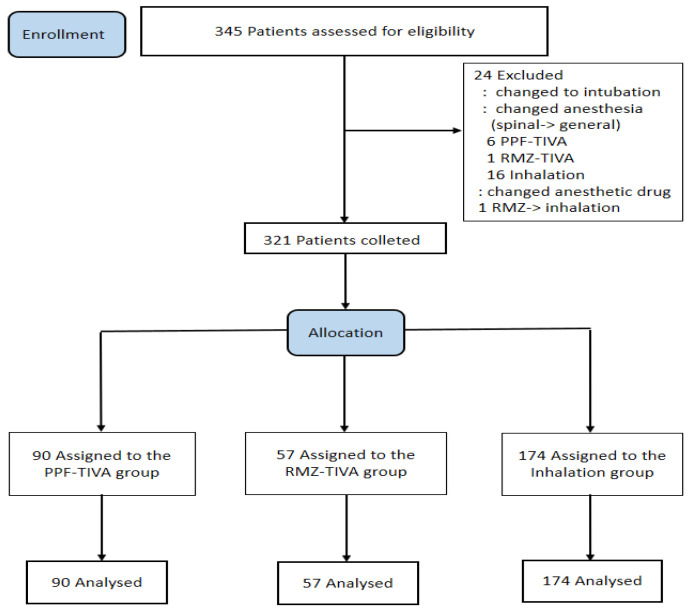
Consolidated Standards of Reporting Trials (CONSORT) flow diagram.

A total of 345 patients were included in this study. Twenty-four patients were excluded according to the exclusion criteria. After excluding these patients, 321 were finally included in the analysis (Figure 1).

The incidence rate of rocuronium use during anesthetic induction was 28.3%. We categorized the anesthesia group into three groups for analysis. The three groups included PPF-TIVA, RMZ-TIVA, and inhalation groups. The patient characteristics in the three groups were similar (Table 1).

Patients in the PPF-TIVA group showed a lower incidence of rocuronium use during anesthetic induction and surgery than those in the other two anesthetic groups (PPF-TIVA: 24.4% vs. RMZ-TIVA: 59.6% vs. Inhalation: 48.3%, *p* = 0.003). During anesthetic induction, the incidence of rocuronium use was also the lowest in the PPF-TIVA group (PPF-TIVA: 14.0% vs. RMZ-TIVA: 36.8% vs. inhalation: 32.7%, *p* = 0.002). As a result of post hoc analysis, the incidence of rocuronium use during the induction period was lower in the PPF–TIVA group when compared with either the RMZ or inhalation groups. (PPF-TIVA vs. RMZ-TIVA, *p* = 0.002, PPF-TIVA vs. inhalation, *p* = 0.001.) There was no difference in the incidence of rocuronium use during anesthetic induction time between the RMZ and inhalation groups. (RMZ-TIVA vs. inhalation, *p* = 0.57.) There were no statistically significant differences in the total amounts of rocuronium used per patient among the three groups. However, according to the results of post hoc analysis, a larger amount of rocuronium was used in the RMZ group than in the other two groups at the start of surgery. (PPF-TIVA vs. RMZ-TIVA, *p* = 0.001, RMZ-TIVA vs. inhalation, *p* = 0.04, PPF-TIVA vs. Inhalation, *p* = 0.111.) (Table 2).

Midazolam premedication and short anesthetic time were associated with lower incidences of rocuronium use. Other variables did not affect the incidence of rocuronium use (Table 3).

## 4. Discussion

In this retrospective study, we analyzed data from 321 patients with a mean age of approximately 50 years who underwent elective minor orthopedic surgery under general anesthesia at a single medical institution.

The results of this study showed differences in the incidences of rocuronium use among the anesthesia groups. Furthermore, midazolam as premedication decreased rocuronium use. In addition, rocuronium was used more frequently with prolonged duration of anesthesia.

The insertion of the SGA device during anesthetic induction requires a sufficient depth of anesthesia in order to suppress airway reflexes and to secure adequate jaw relaxation. PPF is a common intravenous anesthetic agent used for SGA device insertion owing to its significant depressant effect on airway reflexes [7]. Sevoflurane is suitable for inhalational induction technique in high concentrations because of its low blood–gas solubility and minimal respiratory irritant effects [8]. Sevoflurane took a longer time than PPF for jaw relaxation and SGA device insertion, but the hemodynamics were maintained better with sevoflurane than with PPF [9]. Both PPF and sevoflurane are potent bronchodilators; the former acts through a reduction in parasympathetic tone, while the latter predominantly acts through a reduction in serotonin 5-hydroxy-tryptamine receptor activity and inhibition of adenosine 5′-triphosphate (ATP)-induced contraction of bronchial smooth muscle. However, PPF was superior to sevoflurane in suppressing laryngeal reflex responses. PPF appears to be superior to inhalational anesthetics in terms of airway dynamics during SGA insertion [10].

SGA device insertion conditions improve when PPF is used in combination with drugs, such as midazolam, fentanyl, lidocaine, and succinylcholine [11,12].

Benzodiazepines (BZDs) cause anxiolysis, sedation, and amnesia without any analgesic effects. Midazolam (onset, 1~3 min; duration, 15~60 min) is commonly used as a premedication. Midazolam is an effective premedication drug in children that acts synergistically with PPF to reduce the effective dose required for SGA device insertion [13]. The application of midazolam before the induction drug effectively blunted the airway reflexes and adequately facilitated SGA device insertion. Midazolam may cause upper airway obstruction; however, it remains unclear whether it has a dose-dependent effect [14].

RMZ is a rapidly metabolized BZD that has been shown to be an effective sedative–hypnotic agent for the induction of general anesthesia [15,16]. The SGA device insertion conditions in the RMZ group were non-inferior to those in the PPF group in Tang et al.’s study. However, they reported that the induction time was significantly longer, and transiently elevated plateau airway pressure and laryngospasm occurred more frequently in the RMZ group [17]. In the present study, the incidence of rocuronium use in the RMZ-TIVA group was 59.6%. When rocuronium was used, it was administered mostly during SGA device insertion and at the start of surgery. Considering previous studies and our results, it is conceivable that in the initial injection stage, RMZ may have little effect on reducing the upper airway reflex. However, to date, no studies have reported about upper airway reflexes when RMZ was used. Further research is needed on this subject. A recent study compared PPF and RMZ for general anesthesia using a laryngeal mask. The authors found a greater incidence of body movements and hiccups in the RMZ group [18].

Some studies suggest induction using PPF combined with a short-acting opioid without NMBAs, as it provides safe and fast recovery without the residual effects of NMBAs [19]. The use of NMBAs during surgery may result in a postoperative residual neuromuscular block, which can increase the risk of postoperative pulmonary complications [20]. It has been suggested that when using NMBAs, the potential benefits of NMBAs must always be balanced against the increased risk of postoperative pulmonary complications [21]. Na et al. found that NMBAs did not facilitate SGA insertion, and that the insertion attempt number and insertion time did not differ whether NMBAs were used or not. These findings imply that NMBAs are not necessary for anesthetic induction using SGAs [5]. Although the routine use of NMBAs is not recommended during anesthetic induction with SGAs [22], it seems also true that NMBAs may be helpful for solving airway problems associated with the use of SGA devices [23,24,25]. 

This study has some limitations. First, owing to the retrospective nature of this study, the results may have been affected by confounding factors and selection bias. Relying on medical records, the precise timing of administration was sometimes not clearly documented because of non-uniformity in data collection. Second, the limitations of this study include possible differences in the indications for rocuronium use by the attending anesthesiologist during SGA device insertion. Third, we limited our analysis to incidences of rocuronium use according to anesthetic variables. Therefore, the conclusions should be interpreted conservatively and a prospective study appears to be necessary.

## 5. Conclusions

The incidence of rocuronium use during anesthetic induction with SGA devices was significantly lower with the PPF-TIVA compared to that using RMZ-TIVA or inhalational anesthesia. Premedication with midazolam and shorter operation times were associated with a significantly lower incidence of rocuronium use.

## Figures and Tables

**Table 1:** Demographic profile of the patients in the three subgroups (N = 321).

Characteristic	PPF-TIVA (*n* = 90)	RMZ-TIVA (*n* = 57)	Inhalation (*n* = 174)	*p* Value
SGA profile				
SGA type, *n* (I-gel/Supreme/Flexible)	7/76/7	3/51/3	137/19/18	
SGA size (3/4/5), *n*	43/36/11	21/31/5	86/81/7	
ASA physical status (I/II/III), *n* *	34/48/8	23/30/4	59/91/24	
Demographic variables				
Sex (male/female), *n*	37/53	31/26	74/110	
Age (years) *	53.46 ± 16.83	51.39 ± 16.22	51.76 ± 20.00	0.821
Height (cm) *	162.0 [156.00–172.25]	165.19 ± 8.61	162.00 [155.00–176.00]	0.254
Weight (kg) *	63.50 [53.00–87.00]	62.00 [56.00–83.00]	64.92 ± 13.18	0.971
BMI (kg/m^2^) *	23.98 [21.64–29.53]	22.94 [21.51–26.56]	24.37 ± 3.83	0.594
Anesthesia time (min) *	77.57 ± 37.13	78.39 ± 33.22	87.65 ± 42.20	0.051
Surgery time (min) *	49.89 ± 31.81	51.19 ± 26.65	58.07 ± 36.99	0.097

* Continuous values are shown as the mean ± standard deviation or median [25th–75th interquartile range]. Categorical variables are expressed as the number of patients. ASA, American Society of Anesthesiologists. BMI, Body Mass Index.

**Table 2:** The incidence and amount of rocuronium use during anesthetic induction and surgery.

Group	PPF-TIVA (*n* = 90)	RMZ-TIVA (*n* = 57)	Inhalation (*n* = 174)	*p* Value
Total use, *n* (%)	22 (24.4)	34 (59.6)	84 (48.3)	0.003
Dose (mg) *	9.94 ± 19.61	12.59 ± 14.11	11.15 ± 13.88	0.611
Anesthetic induction, *n* (%)	13 (14.4)	21 (36.8)	57 (32.7)	0.002
(diffi. vent./self/diffi. insert./hiccup) #	(11/2/1/0)	(14/5/1/1)	(31/19/7/0)	
Dose (mg) *	3.39 ± 7.93	7.32 ± 11.83	6.58 ± 10.28	0.023
During surgery				
at start, *n*	6	15	34	
Dose (mg) *	1.00 ± 4.25	4.46 ± 8.51	2.50 ± 5.29	0.002
Thereafter, *n*	6	7	24	
Dose (mg) *	0.89 ± 3.56	1.61 ± 4.58	1.81 ± 4.93	0.290

* Continuous values are shown as the mean ± standard deviation. Categorical variables are expressed as the number of patients. # diffi. vent.: difficult ventilation/self/diffi. insert.: difficult insertion/hiccup.

**Table 3:** Logistic regression of variables associated with rocuronium use.

Variable	Odd Ratio (95% CI)	*p* Value
Demographic variables		
Age, year	1.002 (0.988–1.017)	0.773
Sex		
Male	Reference *	
Female	0.631 (0.346–1.180)	0.153
Height	0.953 (0.823–1.103)	0.515
Weight	1.052 (0.877–1.261)	0.586
BMI, kg/m^2^	0.849 (0.519–1.389)	0.515
Anesthetic variables		
Premedication (midazolam)	0.671 (0.521–0.866)	0.002
Anesthetic induction agent		
PPF-TIVA	as reference *	
RMZ-TIVA	6.832 (3.033–15.388)	0.000
Inhalation	2.298 (1.321–3.997)	0.003
Anesthetic time (min)	1.006 (1.000–1.012)	0.036
Surgical time (min)	0.985 (0.965–1.006)	0.165

* For multiple indicator variables, one indicator is the reference against which the others in that group are compared.

## Data Availability

The raw data supporting the conclusions of this article will be made available by the authors on request.

## References

[1] Singh A., Bhalotra A.R., Anand R. (2018). A comparative evaluation of ProSeal laryngeal mask airway, I-gel and Supreme laryngeal mask airway in adult patients undergoing elective surgery: A randomised trial. Indian J. Anaesth..

[2] Artime A., Hagberg C.A. (2020). Airway Management in the Adult. Miller’s Anesthesia.

[3] Kong M., Li B., Tian Y. (2016). Laryngeal mask airway without muscle relaxant in femoral head replacement in elderly patients. Exp. Ther. Med..

[4] Hemmerling T.M., Beaulieu P., Jacobi K.E., Babin D., Schmidt J. (2004). Neuromuscular blockade does not change the incidence or severity of pharyngolaryngeal discomfort after LMA anesthesia. Can. J. Anaesth..

[5] Na H.-S., Jeon Y.-T., Shin H.-J., Oh A.-Y., Park H.-P., Hwang J.-W. (2015). Effect of paralysis at the time of ProSeal laryngeal mask airway insertion on pharyngolaryngeal morbidities. A randomized trial. PLoS ONE.

[6] Gong Y.-H., Yi J., Zhang Q., Xu L. (2015). Effect of low dose rocuronium in preventing ventilation leak for flexible laryngeal mask airway during radical mastectomy. Int. J. Clin. Exp. Med..

[7] Marik P.E. (2004). Propofol: Therapeutic indications and side-effects. Curr. Pharm. Des..

[8] Forman S.A., Ishizawa Y. (2015). Inhaled anesthetic pharmacokinetics: Uptake, distribution, metabolism, and toxicity. Miller’s Anesthesia.

[9] Koppula R., Shenoy A. (2005). Comparison of sevoflurane with propofol for laryngeal mask airway insertion in adults. J. Anaesthesiol. Clin. Pharmacol..

[10] Yamakage M., Namiki A. (2003). Cellular mechanisms of airway smooth muscle relaxant effects of anesthetic agents. J. Anesth..

[11] Driver I.K., Wiltshire S., Mills P., Lillywhite N., Howard-Griffin R. (1996). Midazolam co-induction and laryngeal mask insertion. Anaesthesia.

[12] Goyagi T., Tanaka M., Nishikawa T. (2003). Fentanyl decreases propofol requirement for laryngeal mask airway insertion. Acta Anaesthesiol. Scand..

[13] Bhaskar P., Malik A., Kapoor R., Kohli M., Agarwal J., Harjai M. (2010). Effect of midazolam premedication on the dose of propofol for laryngeal mask airway insertion in children. J. Anaesthesiol. Clin. Pharmacol..

[14] Ehsan Z., Mahmoud M., Shott S.R., Amin R.S., Ishman S.L. (2016). The effects of anesthesia and opioids on the upper airway: A systematic review. Laryngoscope.

[15] Chae D., Kim H.-C., Song Y., Choi Y.S., Han D.W. (2022). Pharmacodynamic analysis of intravenous bolus remimazolam for loss of consciousness in patients undergoing general anaesthesia: A randomised, prospective, double-blind study. Br. J. Anaesth..

[16] Doi M., Morita K., Takeda J., Sakamoto A., Yamakage M., Suzuki T. (2020). Efficacy and safety of remimazolam versus propofol for general anesthesia: A multicenter, single-blind, randomized, parallel-group, phase IIb/III trial. J. Anesth..

[17] Tang S., Lu J., Xu C., Wei L., Mei S., Chen R., Meng Q.-T. (2023). Feasibility and Safety of Remazolam versus Propofol When Inserting Laryngeal Masks Without Muscle Relaxants During Hysteroscopy. Drug Des. Dev. Ther..

[18] Yang C., Jiao J., Nie Y., Shao W., Zhang H., Huang S. (2024). Comparison of the bispectral indices of patients receiving remimazolam and propofol for general anesthesia: A randomized crossover trial. Anaesth. Crit. Care Pain Med..

[19] Bettelli G. (2006). Which muscle relaxants should be used in day surgery and when. Curr. Opin. Anaesthesiol..

[20] Murphy G.S., Szokol J.W., Avram M.J., Greenberg S.B., Shear T.D., Vender J.S., Parikh K.N., Patel S.S., Patel A. (2015). Residual neuromuscular block in the elderly: Incidence and clinical implications. Anesthesiology.

[21] Kirmeier E., Eriksson L.I., Lewald H., Fagerlund M.J., Hoeft A., Hollmann M., Meistelman C., Hunter J.M., Ulm K., Blobner M. (2019). Post-anaesthesia pulmonary complications after use of muscle relaxants (POPULAR): A multicentre, prospective observational study. Lancet Respir. Med..

[22] Díaz-Cambronero O., Serrano A., Abad-Gurumeta A., Martinez I.G., Esteve N., Alday E., Ferrando C., Mazzinari G., Vila-Caral P., Oyonarte C.E. (2023). Perioperative neuromuscular blockade. 2020 update of the SEDAR (Sociedad Española de Anestesiología y Reanimación) recommendations. Rev. Española Anestesiol. Reanim. (Engl. Ed.).

[23] Fujiwara A., Komasawa N., Nishihara I., Miyazaki S., Tatsumi S., Nishimura W., Minami T. (2015). Muscle relaxant effects on insertion efficacy of the laryngeal mask ProSeal^®^ in anesthetized patients: A prospective randomized controlled trial. J. Anesthesia.

[24] Hattori K., Komasawa N., Miyazaki Y., Kido H., Deguchi S., Minami T. (2016). Muscle relaxant facilitates i-gel insertion by novice doctors: A prospective randomized controlled trial. J. Clin. Anesth..

[25] Fujimoto M., Kubota F., Yamamoto T. (2020). The effect of rocuronium on ventilatory leak and sealing pressure using a supraglottic airway device: A randomized clinical trial. Acta Anaesthesiol. Scand..

